# The role of positive and negative affective response to acute exercise in physical activity engagement

**DOI:** 10.1002/alz70860_104611

**Published:** 2025-12-23

**Authors:** Marta Stojanovic, Thinh Huynh, Maria DelGiudice, John C. Morris, Denise M Head

**Affiliations:** ^1^ Washington University in St. Louis, St. Louis, MO, USA; ^2^ Washington University in St. Louis, School of Medicine, St. Louis, MO, USA; ^3^ Knight Alzheimer Disease Research Center, St. Louis, MO, USA

## Abstract

**Background:**

Promoting physical activity in later adulthood is essential for facilitating greater health and potentially reducing the risk of developing Alzheimer disease (AD). A more positive affective response to acute exercise has been linked to greater physical activity. However, previous studies primarily focused on younger adults and did not use affective circumplex to assess affect. Hence, the first goal was to examine the role of both positive and negative affect during exercise, as well as high arousal positive (HAP), low arousal positive (LAP), high arousal negative (HAN), and low arousal negative (LAN) affect after acute exercise, in physical activity levels in middle‐aged and older adults. Additionally, age and accumulation of AD pathology have been linked to reduced physical activity levels. Hence, the second goal was to examine whether age and amyloid levels moderate the association between affective response to exercise and physical activity levels.

**Method:**

Cognitively normal individuals from the Knight ADRC Adult Children Study (*n* = 122; age range 44‐85 years) completed an acute bout of moderate‐intensity exercise. Affect during was measured every 5 minutes, while affect post‐exercise was measured at baseline, immediately after, 15 minutes, and 30 minutes post‐exercise. Participants also completed one week of actigraphy, which objectively measured their physical activity levels. Finally, amyloid pathology was estimated with positron emission tomography (PET).

**Result:**

Affective response during exercise was not associated with physical activity levels (*p* = 0.119). Changes in HAP and HAN affect post‐exercise did not predict physical activity (*p*'s>0.158). There was a trend for a greater decrease in LAN affect (*p* = 0.057) to be associated with more physical activity. A greater increase (*p* = 0.002) and a quicker return to baseline (*p* = 0.024) of LAP affect was associated with more physical activity at younger ages. Finally, PET‐Amyloid was not a significant moderator (*p*'s>0.156).

**Conclusion:**

These results suggest that changes in low arousal affect in response to exercise may be more sensitive predictors of physical activity in later adulthood. These findings can inform future interventions aimed at enhancing affective responses to promote physical activity. While amyloid may have limited utility as a moderator, future studies will investigate whether tau biomarkers can significantly impact these effects.

